# The Prevalence of Blinding Trachoma in Northern States of Sudan

**DOI:** 10.1371/journal.pntd.0001027

**Published:** 2011-05-31

**Authors:** Awad Hassan, Jeremiah M. Ngondi, Jonathan D. King, Balgesa E. Elshafie, Ghada Al Ginaid, Mazin Elsanousi, Zeinab Abdalla, Nabil Aziz, Dieudonne Sankara, Victoria Simms, Elizabeth A. Cromwell, Paul M. Emerson, Kamal H. Binnawi

**Affiliations:** 1 Federal Ministry of Health, Khartoum, Sudan; 2 The Carter Center, Atlanta, Georgia, United States of America; 3 Department of Public Health and Primary Care, University of Cambridge, Cambridge, United Kingdom; 4 The Carter Center, Khartoum, Sudan; University of California San Francisco, United States of America

## Abstract

**Background:**

Despite historical evidence of blinding trachoma, there have been no widespread contemporary surveys of trachoma prevalence in the northern states of Sudan. We aimed to conduct district-level surveys in this vast region in order to map the extent of the problem and estimate the need for trachoma control interventions to eliminate blinding trachoma.

**Methods and Findings:**

Separate, population based cross-sectional surveys were conducted in 88 localities (districts) in 12 northern states of Sudan between 2006 and 2010. Two-stage cluster random sampling with probability proportional to size was used to select the sample. Trachoma grading was done using the WHO simplified grading system. Key prevalence indicators were trachomatous inflammation-follicular (TF) in children aged 1–9 years and trachomatous trichiasis (TT) in adults aged 15 years and above. The sample comprised 1,260 clusters from which 25,624 households were surveyed. A total of 106,697 participants (81.6% response rate) were examined for trachoma signs. TF prevalence was above 10% in three districts and between 5% and 9% in 11 districts. TT prevalence among adults was above 1% in 20 districts (which included the three districts with TF prevalence >10%). The overall number of people with TT in the population was estimated to be 31,072 (lower and upper bounds = 26,125–36,955).

**Conclusion:**

Trachoma mapping is complete in the northern states of Sudan except for the Darfur States. The survey findings will facilitate programme planning and inform deployment of resources for elimination of trachoma from the northern states of Sudan by 2015, in accordance with the Sudan Federal Ministry of Health (FMOH) objectives.

## Author Summary

Trachoma is an infectious disease which is caused by a bacterium, *Chlamydia trachomatis* and is the leading cause of preventable blindness, estimated to be responsible for 2.9% of blindness globally. The World Health Organization (WHO) recommends an integrated strategy for control and elimination of blinding trachoma known as SAFE, which stands for: surgery; antibiotics; facial cleanliness; and environmental improvement. In order to identify districts where trachoma is a public health problem, we undertook 88 district-level surveys in 12 northern states of Sudan. Our findings revealed that interventions to prevent blinding trachoma are recommended in 14 out of 88 districts where the prevalence of trachomatous inflammation-follicular (TF) in children aged 1–9 years exceeded the WHO thresholds for intervention. Services to provide surgery to those with trachomatous trichiasis (TT) should be prioritized in 20 districts where prevalence of TT in adults exceeded 1%. These findings are important since they will help the Sudan Federal Ministry of Health (FMOH) to prioritize resources for elimination of trachoma.

## Introduction

Trachoma is an eye disease caused by ocular infection with *Chlamydia trachomatis*, which can result in blindness after cycles of repeated infections. The World Health Organization (WHO) estimates that trachoma accounts for 2.9% of blindness globally [1]. Since 1997, the WHO has advocated for the ‘SAFE’ strategy (Surgery, Antibiotics, Facial hygiene and Environmental improvement) for trachoma control and elimination of blinding trachoma [2]. Implementation of SAFE is targeted at the district level with thresholds of disease prevalence used to determine which districts qualify for intervention. Population based prevalence surveys are the “gold standard” for estimating prevalence of the clinical signs of trachoma in populations and are therefore essential for programme planning, implementation, monitoring and evaluation [3].

Trachoma has long been known to be prevalent in parts of the Sudan. A report by MacCallan in 1934 documented trachoma among school pupils in Khartoum and further north among school children in Nubia (North of Wadi Halfa) [4]. Surveys undertaken by the WHO in the Northern Province between 1963 and 1964 in Atbara Town and surrounding villages revealed trachoma to be a serious public health problem [5]. In 1975, a review of records dating from 1959 to 1969 reported the highest rate of trachoma in the Northern Province and suggested a reducing gradient as one moved further southwards [6]. In addition, the 1975 study also surveyed children aged 0–15 years in Atbara Town and revealed findings similar to those reported a decade earlier by Majcuk [5]. While this evidence demonstrates the historical presence of trachoma in Sudan, these earlier studies used trachoma diagnostic criteria which differ from the current WHO simplified grading system [7], and reflect a pattern of disease that may no longer be relevant.

A survey of 14 villages in Wadi Halfa (Northern State) in 2000 revealed that prevalence of trachomatous inflammation follicular (TF) and/or trachomatous inflammation intense (TI) was 47% among children aged 1–10 years while 4% of women aged over 40 years had trachomatous trichiasis (TT); confirming trachoma as a serious public health problem according to the WHO standards [8]. Despite the historical evidence of trachoma in northern Sudan, there had been no large scale surveys to map trachoma prevalence at the district level in this vast region. This study aimed to assess the northern states of Sudan using contemporary trachoma survey methods in order to estimate the need for trachoma control interventions and plan for elimination of trachoma in the region.

## Methods

### Ethical statement

The surveys were a routine public health practice to inform implementation of SAFE interventions. We used verbal informed consent which is routine practice during surveys undertaken by National Trachoma Control Programs. The Institutional Review Board of Emory University (IRB # 079-2006) and the Sudan Federal Ministry of Health approved the survey protocol and verbal consent procedures. Verbal informed consent to participate was given by the head of the household, each individual and parents of children in accordance with the declaration of Helsinki. Consent for household interviews and trachoma examination was documented by interviewers and examiners on the data collection forms. Personal identifiers were removed from the data set before analyses were undertaken.

### Study site

**Figure 1:**
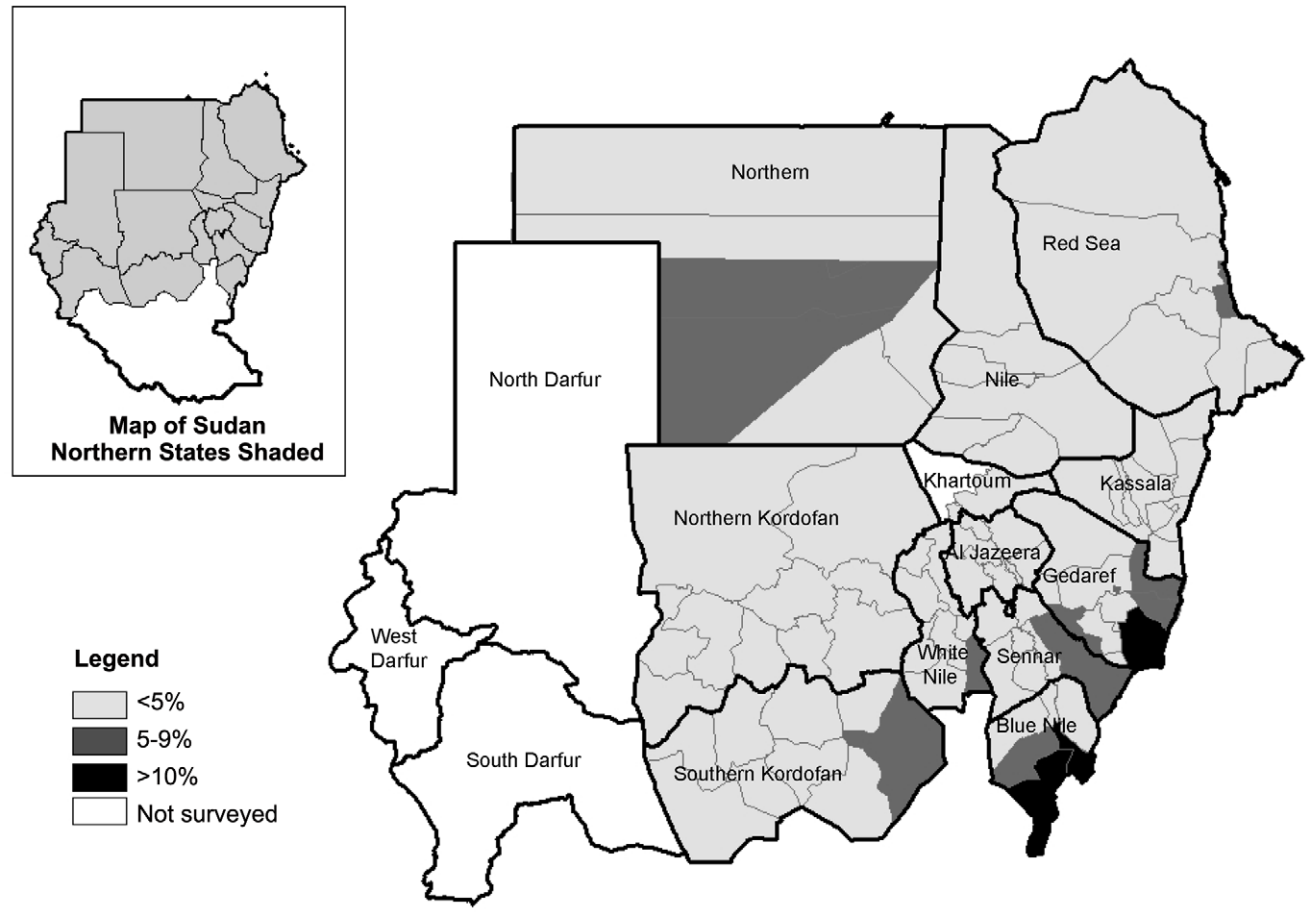
Map of Sudan showing the prevalence of inflammation-follicular (TF) in children aged 1–9 years.

Sudan is the largest country in Africa covering an area of 2.5 million square kilometres. The survey was undertaken in 88 localities (districts) from 2006 to 2010, which together compose 12 out of 15 northern states of Sudan (Figure 1, Map). It was not possible to conduct population-based probability sampling in the three states in the Darfur region (34 districts total) due to internal migration and security concerns.

### Sampling

The sample size was calculated to allow for estimation of at least 10% prevalence of trachomatous inflammation follicular (TF) in children aged 1–9 years within a precision of 5% given a 95% confidence limit and a design effect of 3. We also aimed to estimate at least 3% prevalence of trachoma trichiasis (TT) in persons aged 15 years and above within a precision of 2% at 95% confidence limit and a design effect of 2. Additionally we assumed a 10% non-response rate. Therefore at least 456 children aged 1–9 years and 614 persons aged 15 years and above were to be examined per district. In each district, a two-stage cluster random sampling design with probability proportional to size was used to select the sample. A cluster was defined as the smallest administrative area (i.e. a village in the rural districts or recognised administrative units in the urban districts). A line list (sampling frame) of the names and estimated populations of all clusters in the district was prepared. In the first stage, clusters were randomly selected with probability proportional to the estimated population using computer generated random numbers. Fifteen clusters were selected at random in each district; however, fewer clusters (six) were selected in eight districts comprising densely populated urban areas. In the second stage, 20 households were selected from each cluster using the mapping and segmentation method [9]. All residents of selected households were identified by the heads of household and enumerated by the survey teams. Eligible participants who were present underwent eye examination. An attempt was made to examine absentees by returning to households where people were absent on the day of the survey. It was not possible to return to the village on a different day to follow-up any absentees due to logistical constraints.

### Trachoma grading

Examination for trachoma signs was conducted by doctors and ophthalmic medical assistants trained in using the WHO simplified grading system [7]. Potential examiners underwent training to apply the simplified grading scheme led by an ophthalmologist experienced in trachoma grading. A reliability study was conducted using a set of standardised photographs and an additional reliability study of 50 patients was performed at each training. Examiners had to achieve at least 80% inter-observer agreement in identifying trachoma signs compared to the ophthalmologist to participate in the survey.

All eligible household residents present on the day of the survey were invited to undergo eye examination. Prior to screening for signs of trachoma, faces of children were briefly inspected for cleanliness and defined as “clean” if nasal and/or ocular discharge were absent. Participants were examined for trachoma signs using a ×2.5 magnifying binocular loupe and torch if the ambient light was insufficient. Each eye was examined first trachomatous trichiasis (TT, defined as the presence of at least one eyelash rubbing on the eyeball or evidence of recent removal of in-turned eyelashes), and the cornea was then inspected for corneal opacities (CO). The upper conjunctiva was subsequently examined for inflammation (TF, and TI) and scarring (TS). Both eyes were examined and findings for the worst affected eye recorded. Signs had to be clearly visible in accordance with the simplified grading system in order to be considered present. Alcohol-soaked cotton-swabs were used to clean the examiner's fingers between examinations. Individuals with signs of active trachoma (TF and/or TI) and residents within the same household were offered free treatment with antibiotics according to national guidelines. TT patients were referred to the health system where free surgery was available.

### Household interviews and observations

Structured interviews with adult household respondents and observations were used to assess demographic and household characteristics. Interviews were conducted by trained local health volunteers under supervision by experienced health officers. Prior to the survey, the questionnaire was translated and printed in Arabic language. The questionnaire was then pilot-tested in a non-survey cluster to standardise interviews, observations and completion of the pre-coded answers.. During household interviews, respondents were asked about: source of drinking water and walking time to fetch water; frequency of washing faces of children; sanitation facilities; and livestock, radio and television ownership. In households reporting latrine ownership, the presence of the latrine was verified by observation. Improved water sources were defined according to the WHO/UNICEF Joint Monitoring Programme (JMP) for Water Supply and Sanitation categories (http://www.wssinfo.org/en/definitions-methods/watsan-categories); and included piped water, borehole, protected dug well, protected spring and rainwater.

### Statistical analysis

Statistical analysis was conducted using Stata 8.2 (Stata Corporation, College Station, Texas). Descriptive statistics were used to examine the sample characteristics and the prevalence of trachoma signs. Confidence intervals for the point estimates were derived using the Huber/White sandwich estimator of variance to adjust for the clustering effects of trachoma. We investigated household factors associated with active trachoma by comparing households where one or more children aged 1–9 years had been diagnosed with TF and/or TI with households where no children had TF and/or TI. Univariate logistic regression analysis was conducted for each potential explanatory factor. Multivariable analysis was then undertaken by stepwise regression analysis for model selection. This involved starting with a null model then proceeding in a sequential fashion of adding/deleting explanatory variables if they satisfied the entry/removal criterion which was set at 5% significance level using a log-likelihood ratio test. To derive estimates of the total number of people with TT, prevalence of TT was adjusted for age and sex according to the population structure. The 95% confidence intervals of the adjusted TT prevalence estimates were multiplied by the population estimates to derive the lower and upper bounds of those requiring TT surgery. Finally, based on the survey findings, we estimated the targets for latrine construction by calculating the number of household latrines required to halve the proportion of households that did not have access to a latrine (millennium development goal [MDG] indicator 7.9) [10].

## Results

### Characteristics of the study population

Table 1 summarises the sample, participants and household characteristics by locality (district). The survey was undertaken in 88 districts and the sample comprised 1,260 clusters from which 25,624 households were surveyed. A total of 106,697 participants (out of the 130,700 enumerated, a response rate of 81.6%) were examined for trachoma signs. Of the 24,003 participants not examined, 88.3% were absent during the household visit and majority (69.1%) were male. Of the participants included in the analysis the mean age was 20.9 (standard deviation [sd] = 19.1) and males comprised 42.0%.

**Table 1:** Characteristics of the sample population by district.

States	Locality	Sample		Participants			Proportion of households (%)						
		Number of clusters	Houses Surveyed	Number of participants	Proportion male (%)	Number of people per HH Mean (SD)	Improved water source	Time to collect water ≤30 minutes	Wash Faces ≥2 times per day	Own pit latrine	Own Livestock	Own radio	Own Television
Northern	Dalgo	15	300	1,120	38.4	5.0 (2.2)	97.7	90.3	70.3	38.0	82.3	72.0	47.3
	Dongola	6	182	893	36.4	5.7 (2.5)	0.0	98.9	53.3	92.3	58.2	76.9	73.6
	El Dabbah	15	300	1,207	42.5	6.2 (2.7)	91.3	93.0	64.0	72.3	82.0	68.7	53.3
	Halfa	15	300	1,089	38.0	5.0 (2.3)	91.7	88.7	60.9	61.0	71.3	59.3	64.0
	Merawi	15	300	1,229	40.5	5.7 (2.6)	92.3	93.7	63.0	93.3	79.9	73.8	79.0
River Nile	Abu Hamad	15	300	1,213	47.7	4.7 (2.0)	22.0	76.0	72.7	71.3	89.0	71.0	59.7
	Atbra	15	300	1,276	42.9	5.3 (2.2)	97.7	100.0	61.3	100.0	20.0	61.3	86.3
	Barber	15	300	1,153	40.4	4.4 (1.9)	100.0	99.7	62.7	98.3	53.7	59.3	69.3
	Eldamar	15	300	1,448	46.3	5.2 (2.3)	94.0	93.3	55.7	85.7	76.3	67.7	48.3
	Elmatama	15	300	1,160	43.8	4.8 (1.9)	93.0	88.3	65.0	87.3	76.7	55.3	49.7
	Shendi	15	300	1,280	40.2	5.4 (2.0)	94.0	94.0	74.7	92.3	62.0	30.7	69.0
Red Sea	Ageeg	15	300	963	40.4	4.2 (1.8)	68.3	83.0	83.7	15.3	92.7	15.3	0.3
	Gabeet El Ma'adin	15	300	1,036	43.3	4.3 (2.0)	0.0	6.7	66.2	1.3	97.7	16.3	13.0
	Halayeb	15	300	885	48.1	3.2 (1.7)	0.0	83.3	53.7	6.3	88.0	5.0	6.7
	Haya	15	300	1,042	42.1	4.2 (1.8)	20.7	36.7	40.1	4.7	91.0	4.0	4.0
	Port Sudan	15	300	1,038	41.3	4.5 (1.8)	20.3	45.0	59.0	67.3	20.7	44.7	51.3
	Dordeeb	15	300	931	35.9	4.2 (1.9)	41.7	37.0	36.7	34.7	63.7	14.7	24.0
	Elginab	15	300	968	42.3	3.9 (2.0)	32.3	52.0	58.0	19.7	85.0	11.0	9.7
	Sawaken	15	300	1,078	39.7	4.9 (2.2)	1.3	67.0	73.3	35.3	72.7	10.3	16.3
	Sinkat	15	300	979	39.4	4.0 (1.6)	18.3	64.7	66.3	23.0	69.3	14.4	16.7
	Tokar	15	300	1,006	39.0	3.6 (1.8)	46.7	21.7	64.3	28.0	80.3	17.7	7.7
Kassala	Hamashkorieb	15	300	790	35.7	3.7 (1.5)	20.0	52.3	46.0	12.0	70.0	0.0	0.0
	Kassala rural	6	178	980	40.7	6.5 (3.2)	0.0	65.0	59.6	50.0	75.4	42.1	10.7
	Kassala urban	6	181	900	43.1	7.2 (2.8)	0.0	83.4	77.2	88.4	39.4	65.7	65.2
	Refi Halfa Eljadidah	15	300	1,241	39.4	5.1 (2.2)	63.0	65.0	39.0	77.7	77.0	71.7	63.7
	Refi Nahr Attbara	15	300	1,270	46.9	4.4 (1.7)	13.7	13.3	76.3	19.7	93.0	28.0	9.0
	Rifi Aroma	15	300	1,046	36.2	4.8 (2.5)	30.3	23.3	63.2	22.7	69.3	22.3	8.3
	Rifi Elgirba	15	300	1,191	40.8	4.9 (2.0)	60.0	92.7	74.7	51.5	67.9	51.7	35.3
	Shemal Eldalta	15	300	835	38.4	3.7 (2.0)	29.0	57.3	67.7	56.0	36.7	14.7	7.0
	Talkok	15	300	1,203	52.3	4.0 (1.5)	23.0	12.3	29.0	11.3	86.0	0.3	0.0
	Wad El Hilio	15	300	1,046	42.6	4.2 (1.8)	21.0	57.7	53.3	28.7	73.0	24.0	6.3
Gedaref	Albutana	15	300	1,369	45.7	4.9 (2.0)	0.3	54.0	81.3	6.3	94.0	41.0	0.0
	El Fashga	15	300	1,281	43.2	5.3 (2.7)	28.0	91.0	68.7	37.7	77.3	63.7	9.3
	El Faw	15	300	1,361	45.0	5.6 (2.2)	49.7	40.0	76.3	27.0	75.3	57.3	18.7
	El Galabat East	15	300	1,420	46.0	5.3 (2.4)	16.0	43.0	76.3	22.0	58.0	55.3	4.0
	El Galabat West	15	300	1,405	43.2	5.5 (2.3)	16.3	96.0	66.3	28.0	71.5	72.3	20.7
	El Rahd	15	300	1,431	44.6	5.0 (2.2)	61.9	83.3	66.2	76.7	64.0	61.3	20.0
	Gadaref Center	15	300	1,263	41.0	5.5 (2.6)	16.7	82.7	45.7	21.3	68.3	63.3	22.3
	Gal Alnahal	15	300	1,335	41.6	5.6 (2.6)	56.7	52.3	72.7	8.3	73.0	68.3	9.3
	Gadaref	15	300	1,449	41.5	5.4 (2.4)	55.3	71.6	74.3	76.0	27.7	63.3	64.3
	Gorisha	15	300	1,373	39.5	5.0 (2.4)	6.7	91.3	78.3	24.0	66.7	47.3	2.7
Khartoum	Jabal Awliya	6	108	719	43.9	8.3 (3.1)	59.3	84.5	75.7	69.4	34.3	74.1	55.6
	Sharg En Nile	6	180	1,116	42.1	8.2 (3.4)	2.8	73.9	86.7	87.2	19.4	64.4	61.1
Gezira	El Hasaheisa	15	300	1,365	40.5	6.0 (2.7)	80.7	77.0	71.7	60.0	69.0	70.3	55.0
	El Kamlin	15	300	1,371	40.5	6.4 (2.9)	79.9	84.3	79.2	64.9	56.2	71.0	59.3
	El Managil	15	300	1,590	41.1	6.5 (2.9)	38.0	64.3	72.8	34.6	82.3	73.7	24.6
	Jnaub El Gezira	15	299	1,470	41.3	6.4 (2.9)	71.6	78.9	72.9	40.1	67.9	72.5	65.2
	Madani Elkubra	15	300	1,414	39.0	6.3 (2.9)	83.0	81.3	67.9	60.5	43.4	72.6	74.8
	Sharg El Gezira	15	298	1,668	41.5	7.1 (3.2)	71.5	86.9	79.5	73.8	66.1	75.8	68.5
	Umm El Gura	15	298	1,600	44.1	7.2 (3.1)	0.7	84.8	75.8	44.0	70.5	64.6	47.5
White Nile	Algetina	15	300	1,438	39.7	5.8 (2.5)	58.7	93.3	61.3	29.0	81.3	62.0	25.1
	Alsalm	15	299	1,073	43.3	4.8 (2.0)	0.0	60.2	34.4	11.0	86.6	46.5	0.3
	Ed Douiem	15	299	1,398	37.3	5.3 (2.2)	30.4	91.0	64.5	32.8	58.2	74.2	41.8
	El Jabalian	15	299	1,285	45.2	6.4 (2.7)	14.0	66.9	78.9	29.1	89.3	67.9	18.4
	Kosti	15	300	1,325	43.7	5.2 (2.1)	18.0	45.3	56.3	21.0	78.3	65.3	16.3
	Omramta	15	300	1,258	37.1	6.5 (2.8)	0.0	88.3	81.7	30.3	92.7	69.7	14.8
	Rabak	15	299	1,534	40.9	5.9 (2.8)	59.2	81.3	63.5	67.6	43.5	34.8	53.7
	Tendelti	15	300	1,277	41.3	5.3 (2.3)	0.0	57.0	75.7	11.7	84.3	61.7	3.3
Sinnar	Abuhojar	15	300	1,415	44.5	5.4 (2.3)	52.3	88.3	70.0	58.3	74.0	51.0	26.3
	Eldali & Elmazmoom	15	300	1,249	46.3	4.5 (2.0)	25.3	44.3	60.7	57.0	76.0	63.7	13.7
	Eldindir	15	300	1,247	41.3	4.8 (2.2)	69.0	95.0	70.7	21.0	75.0	59.7	9.7
	Elsoki	15	300	1,356	41.4	5.2 (2.0)	73.0	82.3	39.5	66.7	65.7	45.3	34.1
	Sennar	15	300	1,216	41.1	5.6 (2.4)	50.3	88.0	65.7	34.0	55.0	66.0	29.3
	Sharg Sinnar	15	299	1,323	43.3	5.4 (2.4)	90.0	80.3	72.2	39.8	85.3	51.2	17.1
	Singa	15	300	1,299	41.0	5.3 (2.2)	62.0	81.3	68.3	70.3	48.8	66.3	60.0
Blue Nile	Baw	10	276	1,435	43.1	7.1 (3.4)	56.5	43.1	73.6	15.2	86.2	42.4	2.2
	Ed Damazin	10	250	1,008	44.8	5.7 (3.3)	20.4	73.2	58.8	56.8	44.8	62.0	34.4
	El Roseires	10	279	1,419	46.2	6.4 (3.7)	14.0	66.7	68.0	68.8	74.2	57.7	9.1
	Geissan	15	300	1,311	46.3	5.6 (2.6)	29.0	58.3	61.7	35.0	73.6	59.3	10.2
	Kurmuk	15	300	1,220	42.7	4.6 (2.1)	70.3	66.3	64.0	25.7	67.0	40.7	0.7
North Kordofan	Abo Zaid	15	300	1,263	40.2	5.1 (2.4)	77.3	80.0	62.0	76.3	93.3	68.0	14.0
	Bara	15	300	1,183	41.5	4.3 (1.9)	34.7	63.3	64.0	42.1	88.3	30.7	8.7
	Elnihood	15	300	1,212	40.5	4.6 (2.1)	14.0	33.0	74.8	88.3	69.3	57.0	20.3
	Ghebeish	15	300	1,266	44.5	4.7 (2.1)	13.7	78.0	46.3	81.3	77.3	70.0	12.0
	Jabrat Elshiekh	15	300	1,260	50.2	4.3 (1.9)	52.3	46.0	56.3	14.3	91.7	17.7	3.0
	Om Roaba	15	300	1,068	40.0	4.1 (1.9)	10.0	58.3	52.0	33.3	70.7	53.3	11.3
	Shikan	15	300	991	40.0	4.3 (1.9)	61.0	89.3	68.0	79.0	31.3	73.7	53.0
	Sowdari	15	300	993	33.3	4.0 (1.9)	26.0	53.3	81.3	46.0	83.0	29.0	5.7
	Wad Banda	15	300	1,124	37.6	4.6 (1.6)	46.7	59.7	65.7	86.3	80.7	42.0	9.3
South Kordofan	Abu Jubaiyeh	15	300	1,302	40.3	4.8 (2.3)	51.3	50.0	72.3	14.3	62.7	26.3	2.0
	Abyei	15	300	1,132	39.8	4.5 (1.8)	7.0	70.3	24.7	69.7	60.7	36.3	16.0
	El Salam	15	300	1,226	40.5	5.3 (2.2)	2.0	88.7	81.3	86.0	53.0	48.7	20.3
	Eldalang	15	300	1,463	48.7	5.4 (2.7)	92.3	64.9	61.2	13.8	68.2	43.1	4.3
	Kadugli	15	300	1,038	39.5	4.0 (1.7)	71.3	97.3	60.7	42.7	48.0	20.0	8.3
	Kaylak	15	300	1,177	44.4	4.0 (1.9)	2.3	66.3	63.3	10.0	89.7	5.0	0.3
	Lagawa	15	300	1,075	36.0	4.1 (1.8)	72.3	76.3	53.7	40.3	77.7	56.0	8.0
	Rashad	15	300	1,134	39.7	4.4 (2.1)	50.0	57.3	76.3	26.3	72.0	31.3	2.0
	Talodi	15	300	1,208	42.8	4.7 (2.1)	79.3	75.7	54.7	11.7	75.3	43.3	2.0

HH household; SD, standard deviation.

Table 1 lists locality level estimates for each household characteristic. Overall, the mean number of people per household was 5.1(sd = 2.5). Overall, household access to an improved water source was 43.1% (range by district 0.0–100) and proportion of households reporting round trip to collect water within 30 minutes was 69.2% (range by district 6.7–100). Washing children's faces at least two times a day was reported in 64.5% (range by district 24.7–88.2) of households. Household latrine ownership was 45.2% (range by district 1.3–100). Proxy indicators of household wealth were: livestock ownership (70.2% [range by district 19.4–97.7]); radio ownership (48.4% [range by district 0.0–76.9]); and television ownership (26.1% [range by district 0.0–86.3]).

### Prevalence of trachomatous inflammation-follicular (TF), clean face and trachomatous trichiasis (TT)

**Figure 2:**
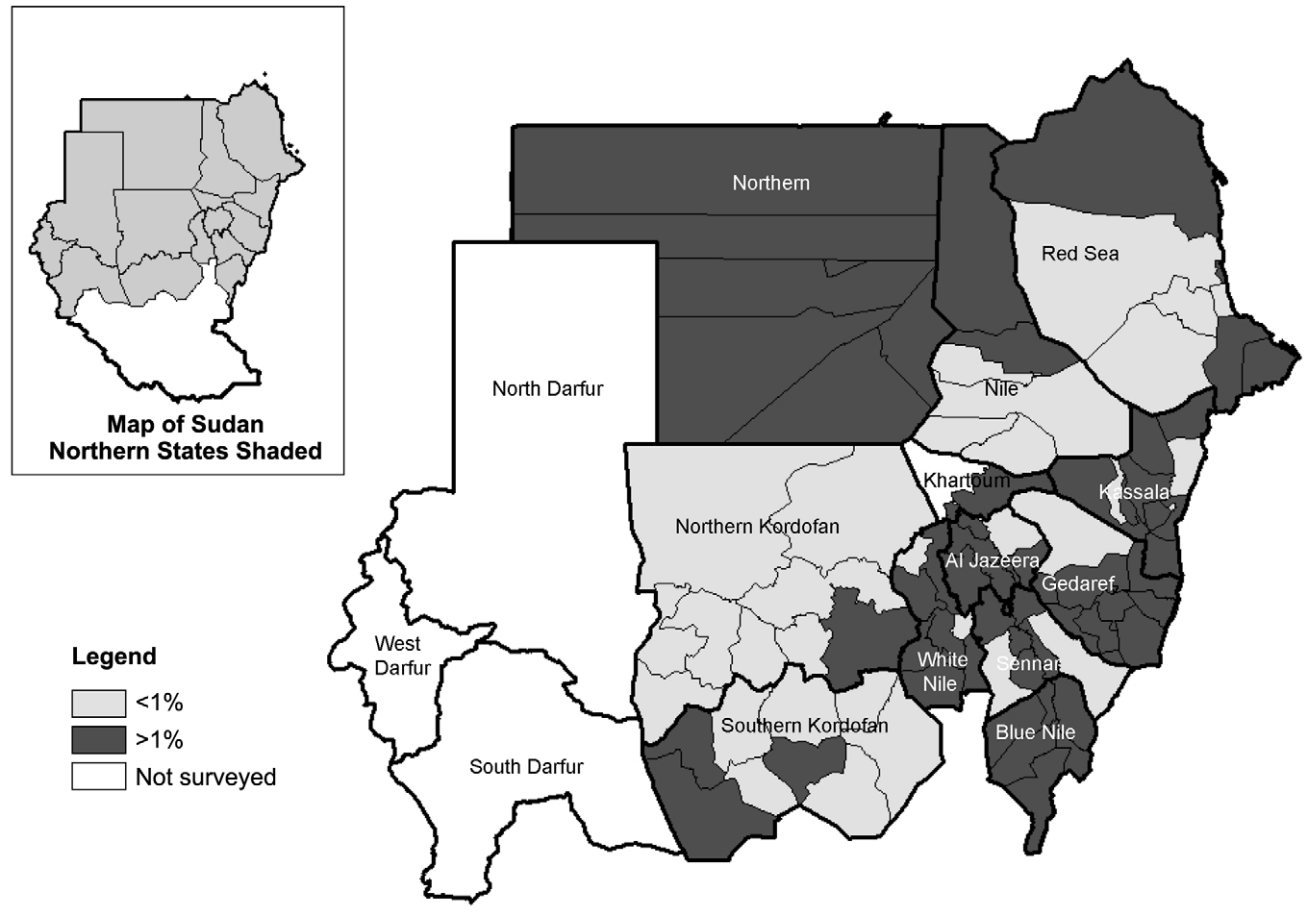
Map of Sudan showing prevalence of trachomatous trichiasis (TT) in adults aged 15 years and above.

**Figure 3:**
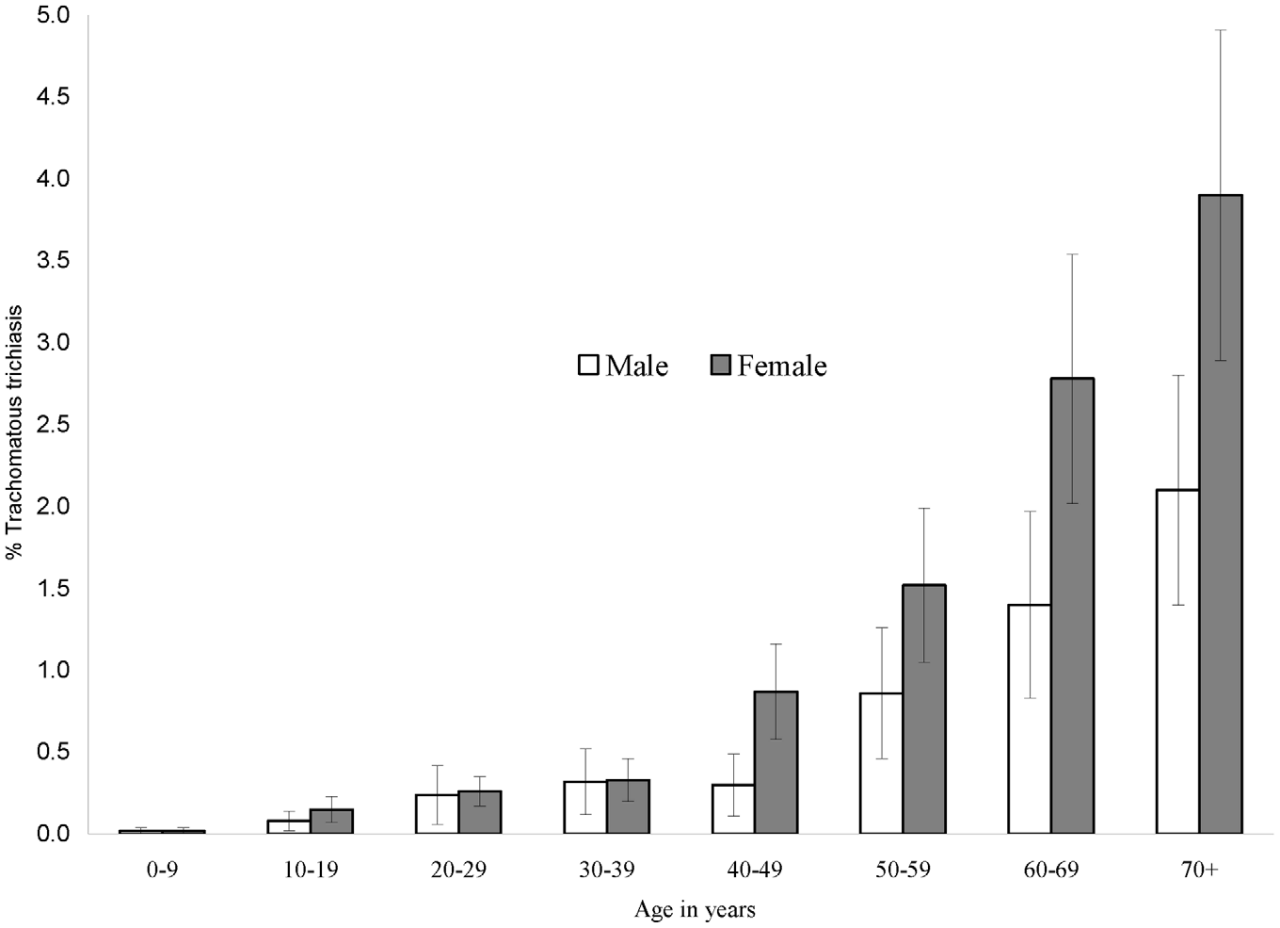
Age-specific prevalence of trachomatous trichiasis (TT) with 95% confidence intervals, by gender.

The prevalence of trachomatous inflammation-follicular (TF), clean face and trachomatous trichiasis (TT) are shown in Table 2 and Figures 1, 2 and 3. The prevalence of TF in children aged 1–9 years by district ranged from 0.0–19.8%. TF prevalence was above 10% in three districts: two in Blue Nile State (Geissan and Kurmuk); and one in Gederaf State (El Galabat East). A total of 11 districts had TF prevalence of between 5 and 9%, including: Dongola in Northern State; Port Sudan and Sawaken in Red Sea State; El Fashga, El Rahd, Gedaref and Gorisha in Gedaref State; El Jabalian in White Nile State; Eldindir in Sinnar State; Baw in Blue Nile State; and Abu Jubaiyeh in South Kordufan State. Overall, 84.7% (range by district 46.9–100) of children aged 1–9 years had a clean face. The prevalence of TT in adults aged 15 years and older by district ranged from 0 to 6.7%. TT prevalence was above the WHO threshold for community based intervention of 1% in 20 districts (which included the three districts with TF prevalence >10%). The prevalence of TT increased with age with an overall significantly higher prevalence among females compared to males (OR [Odds Ratio] = 1.7; 95% CI 1.4–2.2) [Figure 3].

**Table 2:** Prevalence of TF, clean face, TT and SAFE intervention objectives by district.

States	Locality	Children 1–9 years of age			Adults 15 years and above		SAFE intervention objectives			
		Number examined	TF % (95% CI)	Clean face: % (95% CI)	Number examined	TT % (95% CI)	TT cases (Lower & upper bounds)	Antibiotic distribution strategy	Eligible for hygiene promotion	Pit latrines required to meet MDG indicator 7.9
Northern	Dalgo	335	0.3 (0.0–2.1)	80.6 (76.0–84.5)	660	0.9 (0.4–2.0)	106 (91–123)		Yes	1,925
	Dongola	315	**8.6 (5.9–12.2)**	96.5 (93.8–98.1)	497	**1.4 (0.7–2.9)**	757 (646–886)	Targeted	Yes	1,853
	El Dabbah	336	0.3 (0.0–2.1)	86.9 (82.9–90.1)	756	0.7 (0.3–1.6)	270 (230–318)		Yes	2,401
	Halfa	345	0	85.5 (81.4–88.8)	626	**2.4 (1.4–3.9)**	89 (76–104)		Yes	1,116
	Merawi	378	0	96.3 (93.8–97.8)	728	0.8 (0.4–1.8)	445 (380–522)		Yes	1,021
River Nile	Abu Hamad	341	0	93.0 (89.7–95.2)	707	0.6 (0.2–1.5)	176 (149–209)		Yes	1,916
	Atbra	353	0.6 (0.1–2.2)	94.9 (92.1–96.8)	785	0			Yes	0
	Barber	297	0	97.6 (95.1–98.9)	709	**1.1 (0.6–2.2)**	615 (529–716)		Yes	289
	Eldamar	415	0.2 (0.0–1.7)	88.9 (85.5–91.6)	856	0			Yes	2,875
	Elmatama	367	0.3 (0.0–1.9)	95.9 (93.3–97.5)	644	0			Yes	1,331
	Shendi	396	0	93.2 (90.2–95.3)	736	0			Yes	1,590
Red Sea	Ageeg	360	0.3 (0.0–1.9)	90.3 (86.8–92.9)	494	0.2 (0.0–1.4)	137 (116–163)		Yes	4,397
	Gabeet El Ma'adin	383	0	94.8 (92.0–96.6)	522	0			Yes	3,018
	Halayeb	333	0.3 (0.0–2.1)	99.1 (97.2–99.7)	487	0.2 (0.0–1.4)	64 (55–76)		Yes	2,085
	Haya	466	1.1 (0.4–2.6)	86.7 (83.3–89.5)	483	0			Yes	4,951
	Port Sudan	387	**5.4 (3.6–8.2)**	84.8 (80.8–88.0)	554	0.7 (0.3–1.9)	1066 (900–1262)	Targeted	Yes	12,844
	Dordeeb	361	3.3 (1.9–5.8)	87.3 (83.4–90.3)	446	0			Yes	1,508
	Elginab	441	0.9 (0.3–2.4)	77.3 (73.2–81.0)	438	0			Yes	1,782
	Sawaken	449	**6.5 (4.5–9.1)**	84.0 (80.3–87.1)	488	0.2 (0.0–1.4)	95 (79–113)	Targeted	Yes	2,609
	Sinkat	414	4.6 (2.9–7.1)	76.8 (72.5–80.6)	502	0.2 (0.0–1.4)	119 (101–141)		Yes	3,427
	Tokar	407	1.2 (0.5–2.9)	99.0 (97.4–99.6)	539	**1.1 (0.5–2.5)**	165 (139–195)		Yes	4,453
Kassala	Hamashkorieb	365	0	93.7 (90.7–95.8)	341	0.3 (0.0–2.1)	192 (162–227)		Yes	5,915
	Kassala rural	384	0.3 (0.0–1.8)	90.1 (86.7–92.7)	462	**1.1 (0.5–2.6)**	669 (559–800)		Yes	14,708
	Kassala urban	296	0	99.0 (96.9–99.7)	471	0			Yes	2,764
	Refi Halfa Eljadidah	418	0.2 (0.0–1.7)	100.0	664	0.2 (0.0–1.1)	705 (597–832)		Yes	5,746
	Refi Nahr Attbara	469	0	97.4 (95.5–98.5)	652	0.2 (0.0–1.1)	318 (266–379)		Yes	10,774
	Rifi Aroma	417	0.5 (0.1–1.9)	81.8 (77.8–85.2)	506	0.4 (0.1–1.6)	224 (188–266)		Yes	7,127
	Rifi Elgirba	494	0.6 (0.2–1.9)	85.8 (82.5–88.6)	554	0.5 (0.2–1.7)	197 (165–235)		Yes	4,159
	Shemal Eldalta	290	1.0 (0.3–3.2)	83.4 (78.7–87.3)	435	**2.3 (1.2–4.2)**	298 (252–353)		Yes	4,918
	Talkok	538	0.4 (0.1–1.5)	98.5 (97.1–99.3)	611	0			Yes	8,798
	Wad El Hilio	416	2.9 (1.6–5.0)	89.2 (85.8–91.8)	502	0.4 (0.1–1.6)	218 (183–260)		Yes	6,326
Gedaref	Albutana	521	0	100.0	668	0.1 (0.0–1.1)	99 (83–118)		Yes	3,874
	El Fashga	477	**6.1 (4.3–8.6)**	87.2 (83.9–89.9)	602	0.8 (0.3–2.0)	404 (338–483)	Targeted	Yes	10,980
	El Faw	490	3.1 (1.9–5.0)	85.3 (81.9–88.2)	637	0.5 (0.2–1.4)	427 (357–513)		Yes	14,056
	El Galabat East	561	**19.8 (16.7–23.3)**	76.6 (73.0–80.0)	625	**1.9 (1.1–3.3)**	369 (307–443)	Mass	Yes	13,410
	El Galabat West	561	**3.4 (2.2–5.2)**	67.7 (63.8–71.5)	635	**1.3 (0.6–2.5)**	0 (0–0)		Yes	9,146
	El Rahd	709	**7.1 (5.4–9.2)**	71.4 (67.9–74.6)	585	**4.8 (3.3–6.8)**	486 (401–590)	Targeted	Yes	6,235
	Gadaref Center	476	2.7 (1.6–4.6)	85.9 (82.5–88.8)	600	0.5 (0.2–1.5)	202 (169–242)		Yes	7,133
	Gal Alnahal	547	0.9 (0.4–2.2)	71.7 (67.7–75.3)	609	**1.8 (1.0–3.2)**	174 (146–208)		Yes	6,816
	Gadaref	473	**5.9 (4.1–8.4)**	85.4 (81.9–88.3)	753	0.8 (0.4–1.8)	873 (736–1035)	Targeted	Yes	8,158
	Gorisha	638	**8.5 (6.5–10.9)**	81.3 (78.1–84.2)	537	**1.1 (0.5–2.5)**	190 (156–231)	Targeted	Yes	8,379
Khartoum	Jabal Awliya	376	5.1 (3.2–7.8)	63.6 (58.6–68.3)	270	**3.0 (1.5–5.8)**	163 (134–197)		Yes	2,668
	Sharg En Nile	425	3.1 (1.8–5.2)	68.0 (63.4–72.3)	532	**1.1 (0.5–2.5)**	132 (110–158)		Yes	766
Gezira	El Hasaheisa	418	0.2 (0.0–1.7)	86.8 (83.3–89.8)	755	**1.1 (0.5–2.1)**	2124 (1800–2507)		Yes	30,430
	El Kamlin	449	0.2 (0.0–1.6)	93.5 (90.9–95.5)	741	0.9 (0.5–2.0)	2128 (1791–2529)		Yes	29,748
	El Managil	488	2.0 (1.1–3.8)	84.4 (80.9–87.4)	861	**1.9 (1.1–3.0)**	796 (671–943)		Yes	19,999
	Jnaub El Gezira	473	0.4 (0.1–1.7)	81.2 (77.4–84.5)	783	**1.1 (0.6–2.2)**	1539 (1299–1823)		Yes	34,974
	Madani Elkubra	414	0	87.9 (84.4–90.7)	804	0.6 (0.3–1.5)	1316 (1115–1553)		Yes	18,402
	Sharg El Gezira	526	0.8 (0.3–2.0)	88.0 (85.0–90.5)	876	0			Yes	2,733
	Umm El Gura	562	2.5 (1.5–4.2)	77.2 (73.6–80.5)	776	**1.0 (0.5–2.0)**	882 (738–1054)		Yes	21,184
White Nile	Algetina	472	0.4 (0.1–1.7)	89.6 (86.5–92.1)	789	0.6 (0.3–1.5)	517 (436–613)		Yes	14,283
	Alsalm	514	0.4 (0.1–1.5)	72.8 (68.7–76.4)	455	**1.1 (0.5–2.6)**	228 (189–273)		Yes	9,380
	Ed Douiem	560	0.2 (0.0–1.3)	93.4 (91.0–95.2)	637	0.2 (0.0–1.1)	574 (479–687)		Yes	17,683
	El Jabalian	533	**6.4 (4.6–8.8)**	82.6 (79.1–85.5)	586	0.5 (0.2–1.6)	387 (322–465)	Targeted	Yes	12,781
	Kosti	490	0.2 (0.0–1.4)	82.4 (78.8–85.6)	655	0.2 (0.0–1.1)	845 (709–1006)		Yes	27,232
	Omramta	444	0	89.2 (85.9–91.8)	637	0			Yes	6,631
	Rabak	516	0.8 (0.3–2.0)	80.4 (76.8–83.6)	819	0			Yes	7,229
	Tendelti	550	0	68.0 (64.0–71.8)	562	0.2 (0.0–1.3)	259 (216–311)		Yes	10,711
Sinnar	Abuhojar	534	4.5 (3.0–6.6)	83.5 (80.1–86.4)	680	0.9 (0.4–1.9)	304 (255–363)		Yes	5,386
	Eldali and Elmazmoom	442	0.7 (0.2–2.1)	94.8 (92.3–96.5)	664	0.0 (0.0–0.0)			Yes	2,475
	Eldindir	532	**8.5 (6.4–11.1)**	89.1 (86.2–91.5)	538	0.2 (0.0–1.3)	313 (259–377)	Targeted	Yes	12,657
	Elsoki	510	0.6 (0.2–1.8)	84.5 (81.1–87.4)	658	0.5 (0.1–1.4)	524 (438–627)		Yes	7,711
	Sennar	402	0.5 (0.1–2.0)	78.9 (74.6–82.6)	629	0.8 (0.3–1.9)	758 (638–899)		Yes	19,616
	Sharg Sinnar	482	5.0 (3.4–7.3)	82.6 (78.9–85.7)	638	0.5 (0.2–1.4)	553 (463–661)		Yes	14,306
	Singa	447	0.2 (0.0–1.6)	87.9 (84.6–90.6)	665	0.5 (0.1–1.4)	424 (358–502)		Yes	4,734
Blue Nile	Baw	597	8.7 (6.7–11.3)	62.0 (58.0–65.8)	665	0.3 (0.1–1.2)	169 (141–202)	Targeted	Yes	6,485
	Ed Damazin	339	1.5 (0.6–3.5)	84.4 (80.1–87.9)	545	0.7 (0.3–1.9)	275 (231–328)		Yes	4,763
	El Roseires	597	2.2 (1.3–3.7)	77.7 (74.2–80.9)	630	0.8 (0.3–1.9)	166 (138–200)		Yes	2,418
	Geissan	599	**17.4 (14.5–20.6)**	71.5 (67.7–74.9)	556	**6.7 (4.9–9.1)**	154 (127–185)	Mass	Yes	5,092
	Kurmuk	473	**12.3 (9.6–15.5)**	70.8 (66.6–74.7)	543	**4.4 (3.0–6.5)**	273 (227–327)	Mass	Yes	9,393
North Kordofan	Abo Zaid	452	0	79.9 (75.9–83.3)	649	0			Yes	3,027
	Bara	461	2.8 (1.6–4.8)	86.6 (83.1–89.4)	579	0	669 (559–800)		Yes	17,260
	Elnihood	506	1.0 (0.4–2.4)	83.2 (79.7–86.2)	533	0			Yes	2,398
	Ghebeish	541	0.6 (0.2–1.7)	85.4 (82.2–88.1)	554	0			Yes	4,153
	Jabrat Elshiekh	471	1.5 (0.7–3.1)	90.2 (87.2–92.6)	666	0			Yes	5,705
	Om Roaba	415	0.2 (0.0–1.7)	88.0 (84.5–90.7)	526	0.8 (0.3–2.0)	1362 (1148–1616)		Yes	35,834
	Shikan	341	1.8 (0.8–3.9)	88.3 (84.4–91.3)	528	0			Yes	9,997
	Sowdari	451	0.4 (0.1–1.8)	92.2 (89.4–94.4)	421	0			Yes	5,251
	Wad Banda	491	1.4 (0.7–3.0)	90.4 (87.5–92.7)	468	0			Yes	1,530
South Kordofan	Abu Jubaiyeh	605	**6.1 (4.5–8.3)**	82.8 (79.6–85.6)	527	0.2 (0.0–1.3)	410 (340–495)	Targeted	Yes	17,932
	Abyei	479	0	96.0 (93.9–97.5)	507	0.6 (0.2–1.8)	409 (342–489)		Yes	5,478
	El Salam	485	0	85.4 (81.9–88.2)	576	0.3 (0.1–1.4)	224 (186–270)		Yes	1,501
	Eldalang	501	0.6 (0.2–1.8)	46.9 (42.6–51.3)	737	0.1 (0.0–1.0)	585 (490–699)		Yes	21,270
	Kadugli	483	0.2 (0.0–1.5)	85.7 (82.3–88.6)	466	0.2 (0.0–1.5)	594 (496–710)		Yes	15,234
	Kaylak	527	0.2 (0.0–1.3)	100.0	546	0			Yes	3,340
	Lagawa	476	0.4 (0.1–1.7)	92.4 (89.7–94.5)	483	0			Yes	7,258
	Rashad	475	1.1 (0.4–2.5)	75.2 (71.1–78.8)	514	0.2 (0.0–1.4)	501 (417–602)		Yes	17,583
	Talodi	521	0	90.8 (88.0–93.0)	533	0.2 (0.0–1.3)	367 (305–441)		Yes	15,488

MDG, millennium development goal; SAFE, Surgery, Antibiotics, Facial cleanliness, and Environmental improvement; TF, trachomatous inflammation-follicular; TT, trachomatous trichiasis.

The figures in bold show districts with TF prevalence ≥5% and/or prevalence of TT≥1%.

### Household factors associated with active trachoma

Table 3 summarises the univariable and multivariable logistic regression of associations between presence of children with active trachoma in a household and potential risk factors. Univariable analysis showed that increasing household size (OR_[per additional person]_ = 1.2; 95% CI 1.2–1.3), head of household with no formal education (OR = 1.7; 95% CI 1.4–2.1), and keeping livestock within the household compound (OR = 3.0; 95% CI 2.3–4.1) were associated with higher odds of children with active trachoma in a household. On the other hand, reporting washing children's faces 2 or more times a day (OR = 0.7; 95% CI 0.6–0.9); pit latrine ownership (OR = 0.7; 95% CI 0.6–0.9); and television ownership (OR = 0.4; 95% CI 0.3–0.6) were associated with decreased odds of active trachoma. Factors independently associated with increasing odds of active trachoma were: increasing household size (OR_[per additional person]_ = 1.2; 95% CI 1.2–1.3); head of household with no formal education (OR = 1.4; 95% CI 1.1–1.7); and keeping livestock within the household compound (OR = 2.5; 95% CI 01.9–3.7). On the other hand, reporting washing children's faces 2 or more times a day (OR = 0.8; 95% CI 0.6–0.9) and television ownership (OR = 0.4 ; 95% CI 0.3–0.6) were independent predictors of reduced odds of active trachoma.

**Table 3:** Associations of household characteristics and presence of ≥ one children with active trachoma in household.

Household characteristic	Univariate analysis		Multivariate analysis	
	Odds Ratio (95%CI)	p value	Odds Ratio (95%CI)	p value
Increasing household size (per additional person)	1.2 (1.2–1.3)	<0.001	1.2 (1.2–1.3)	<0.001
Head of household with no formal education	1.7 (1.4–2.1)	<0.001	1.4 (1.1–1.7)	0.003
Head of house has heard about trachoma	0.8 (0.6–1.0)	0.068		
Head of house not knowing what causes trachoma	1.1 (0.9–1.3)	0.515		
Improved water source	0.8 (0.6–1.1)	0.184		
Round trip to collect water <30 minutes	0.8 (0.6–1.0)	0.072		
Report of washing children's faces 2 or more times a day	0.7 (0.6–0.9)	0.002	0.8 (0.6–0.9)	0.018
Own pit latrine	0.7 (0.6–0.9)	0.003		
Owning livestock (sheep, cows, goats or camels)	1.1 (0.9–1.4)	0.408		
Keeping livestock in compound	3.0 (2.3–4.1)	<0.001	2.5 (1.9–3.7)	<0.001
Owning radio	0.9 (0.7–1.0)	0.101		
Owning television	0.4 (0.3–0.6)	<0.001	0.4 (0.3–0.6)	<0.001

CI, confidence interval.

### SAFE intervention goals

**Figure 4:**
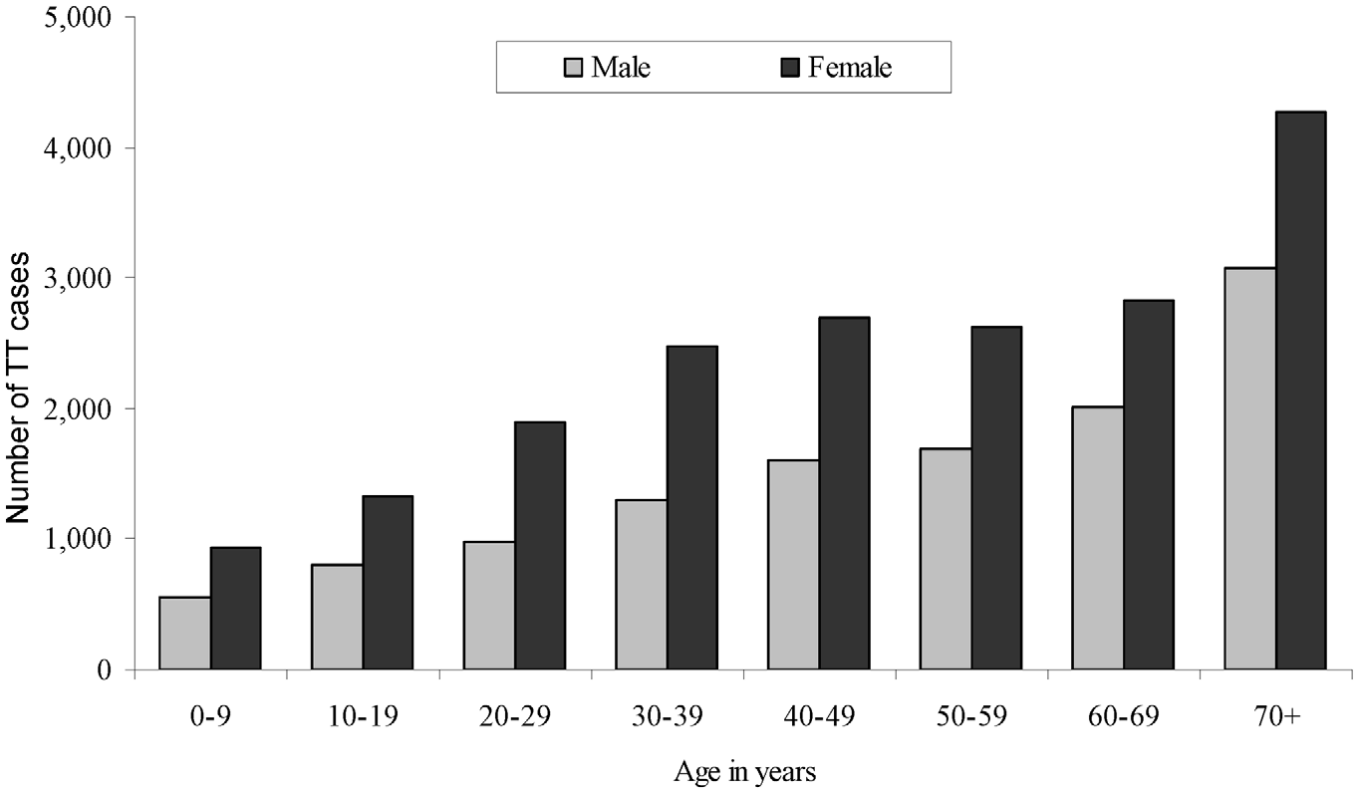
Distribution of estimated cases of trachomatous trichiasis (TT) by age and gender (n = 31,072).

The estimated objectives for the implementation of SAFE in the northern states of Sudan, by locality, are summarised in Table 2. It was estimated that 31,072 people in the northern states had TT (lower and upper bounds = 26,125–36,955) [Figure 4]. Based on TF prevalence estimates, three and 11 districts were eligible for mass antibiotic distribution and targeted antibiotic distribution, respectively. We estimated that all 88 localities surveyed were eligible for facial hygiene promotion while 548,678 household latrines were required to meet the MDG indicator 7.9 in all areas surveyed.

## Discussion

Trachoma surveys are essential for quantifying disease prevalence in order to facilitate programme planning, implementation, monitoring and evaluation. Population-based prevalence surveys are the “gold standard” for estimating prevalence of trachoma in populations. These surveys demonstrate that district-level surveys are feasible to conduct over such a large geographical area district by district and are comparable to surveys in Morocco, The Gambia, and Ethiopia [11]–[13]. This contemporary population-based trachoma prevalence survey covered nearly all of the northern states of Sudan. With the Federal Ministry of Health (FMOH) having set goals to eliminate trachoma from these northern states by the year 2015 [14], these data will be important in establishing health priorities.

These surveys have a number of potential limitations. The desired sample size was obtained in only 56/88 localities. This is largely explained by the pre-survey sample size calculations which assumed 6 persons per household; however, our results revealed a mean household size of 5. In addition the proportion of persons absent from selected households was 16.3% rather than our estimated non-response rate of 10%. Many adult men were absent from the households at the time of the survey team's visit. This may have potentially biased the prevalence of TT in adult men, as healthy men may have been more likely not to be examined while older men may have been more likely to be at home and examined. The number of clusters sampled per district ranged from 6 to 15. Fewer clusters with more households were sampled in the more urban localities since a more pragmatic approach of segmenting the households was required in these densely populated areas. Also, we were not able to survey three states in Darfur region due to security concerns. This limits the ability of the national trachoma program to plan SAFE interventions to reach elimination in the entire northern states. Nonetheless, these areas will require surveying once the security situation improves.

The survey revealed that trachoma is still a public health problem according to the WHO standards in the 3/88 districts where the prevalence of TF in children exceeded 10% and 20/88 districts where the prevalence TT exceeded 1% in adults. In addition, eleven districts had a TF prevalence of between 5 and 9% and were thus eligible for implementation of SAFE with targeted distribution of antibiotics. Household data, specifically latrine ownership, enabled the estimation of the total number of household latrines required to be built in the 88 districts to meet the MDG indicator 7.9 (i.e. reduce the proportion of households without access to sanitation by half) [10].

Identification of risk factors is essential for planning and implementing effective trachoma control programmes. Our risk factor analysis revealed that literacy among household heads, increased frequency of washing children's faces, and proxy indicators of wealth such as livestock and television ownership were associated with a lower prevalence of active trachoma. This supports the need for provision of water and as well as promotion of face hygiene. The results showed that radio and television access were relatively high in most districts, which presents the national program with an opportunity to use state-run media to broadcast trachoma health education and mobilize the population to participate in SAFE interventions.

Compared to previous surveys in the Northern State which showed high prevalence of active trachoma and trichiasis [5], [6] our surveys suggests that active trachoma has declined substantially and trachoma now presents as TT. The distribution of trachoma in the northern states of Sudan appears to be confined to small pockets bordering known endemic areas in Southern Sudan and Ethiopia. Nonetheless, efforts to underpin implementation of the SAFE strategy are required if elimination of trachoma is to be realised. This patchy distribution is a striking contrast to the disease pattern that has been observed in other areas bordering the northern states of Sudan such as Southern Sudan [15] and Amhara Region of Ethiopia [13], where trachoma is still hyper-endemic.

Properly conducted surveys are crucial if the objective of global elimination of blinding trachoma by the year 2020 is to be charted and realised. Our survey used the CRS design advocated by the WHO, to survey vast areas comprising 88 districts in 12 northern states of Sudan. While there are rapid assessment methods used to identify trachoma endemicity, a recent review of survey methods highlighted the benefits of CRS: it is simple; efficient; repeatable; and provides population-based prevalence estimates of all signs of trachoma [3]. Other survey designs that have been proposed for trachoma have limitations. Trachoma rapid assessment (TRA) pitfalls include: non representative sampling; does not estimate prevalence; and lacks consistency and accuracy [16], [17]. Acceptance sampling trachoma rapid assessment (ASTRA) advocates small sample sizes but it is relatively complex, may result in imprecise prevalence estimates and does not estimate cicatricial signs of trachoma [3]. Our survey demonstrates that CRS can be applied on a large scale to provide district level estimates of TF and TT as recommended by the WHO [18].

Our survey revealed that trachoma is a public health problem in nearly a quarter of all districts surveyed. Based on the survey findings, we have estimated intervention objects for the implementation of the SAFE strategy in all areas surveyed. These data are important and will facilitate programme planning and inform deployment of resources for elimination of trachoma from the northern states of Sudan by 2015, in accordance with the FMOH objectives.

## Supporting Information

Checklist S1 STROBE Checklist.
(DOC)
